# Is It Worth Knowing That You Might Die Tomorrow? Revisiting the Ethics of Prognosis Disclosure

**DOI:** 10.3390/clinpract12050084

**Published:** 2022-10-04

**Authors:** Eisuke Nakazawa, Keiichiro Yamamoto, Reina Ozeki-Hayashi, Margie Hodges Shaw, Akira Akabayashi

**Affiliations:** 1 Department of Biomedical Ethics, Faculty of Medicine, University of Tokyo, Tokyo 113-0033, Japan; 2 National Center for Global Health and Medicine, Center for Clinical Sciences, Tokyo 162-8655, Japan; 3 Department of Health Humanities and Bioethics, University of Rochester Medical Center, New York, NY 14642, USA; 4 Division of Medical Ethics, New York University School of Medicine, New York, NY 10016, USA

**Keywords:** prognosis disclosure, clinical ethics, truth disclosure, moralistic viewpoint, human dignity

## Abstract

Ethical discourse on prognosis disclosure is not yet well established. The core of the problem continues to be the dilemma between the right of self-determination and non-maleficence of patients. The prognosis disclosure policy based on Kantian autonomy provides a good solution for the problem. The policy includes demand for strict truth telling and its compatibility with patients’ best interest. However, there remains a discrepancy between theory and practice, especially when prognosis is disclosed just prior to their death. Kantian theory of prognosis is supplemented by a moralistic perspective. The moralistic perspective places high importance on temporality and relationships with others, which all human beings inherently possess. From the moralistic viewpoint, decisions about prognosis disclosure at the final stages of life must be individualized in order to be authentically autonomous. The decision to disclose a prognosis or not can only be determined by the relationships fostered over time with patients.

## 1. Introduction

One of the classic conundrums in biomedical ethics remains truth telling in the medical setting. Although ethical positions on diagnosis disclosure have converged somewhat, the literature on practices around disclosure of prognosis in cancer patients documents a variety of approaches, highlighting continued ethical questions [1,2,3,4]. Stahl and Tomlinson (2017) argue that physician respect for a patient’s desire to not discuss prognosis is a failure of both the principle for respect for autonomy and the principle of beneficence [5]. While this argument is compelling, there remains a discrepancy between theory and practice. Moreover, the arguments are less convincing when prognosis is disclosed to a patient just prior to their death. This paper aims to strengthen the argument for disclosure based on a Kantian autonomy theory by including consideration of timing around disclosure.

## 2. Prognosis Disclosure in Healthcare Settings

A notable gap exists between patient attitudes regarding prognosis disclosure and actual prognosis disclosure [6]. On the one hand, attitude surveys have revealed that many patients desire prognosis disclosure, albeit with cultural differences [7,8,9]. On the other hand, healthcare professionals tend to hesitate about disclosing poor diagnosis to their patients [10]. While the idea of a right of self-determination is increasingly accepted in society, in practice, patients still entrust important health decisions to their family members and healthcare professionals [11,12]. This reliance on medical authority, coupled with a reluctance of physicians to disclose a poor prognosis, results in variations around disclosure practices. According to Daugherty and Hlubocky (2008), only 42% of physicians reported consistently disclosing a terminal prognosis, while 48% disclose the prognosis only when a patient desires disclosure [1]. In addition, 57% of physicians report that they do not always present a detailed clinical time scale [1].

## 3. Prognosis Disclosure and Self-Decision Making

The depth of the ethical discourse on prognosis disclosure remains insufficient [3]. Oncologists continue to report concerns about the impact of disclosure on their patients [13], revealing a lack of resolution around the dilemma between voluntary self-decision making and non-maleficence of patients [14]. Some clinicians make the argument that respect for the right of self-determination requires health care professionals to disclose prognosis only when the patient desires the information. Under this reasoning, prognosis should be disclosed to any patient who desires this; conversely, healthcare professionals should also refrain from disclosing a prognosis if the patient prefers not to know, in accordance with their right not to know. 

Notably, prognosis disclosure opens up the possibility that a patient will experience psychological distress. Studies have identified cultural differences in prognosis disclosure, particularly with regard to how much consideration is given to the principle of non-maleficence when physicians withhold information. For example, O’Kelly found that physicians in the Middle East and Asia tend to withhold diagnoses and prognoses, in accordance with the ‘do no harm’ consideration toward patients [15]. Thus, we consider that, traditionally, a negative atmosphere toward prognostication has been formed. According to the traditional explanation, disclosing a poor prognosis is cruel because it may cause psychological pain to the patient. 

The argument for nondisclosure on the basis of respecting the right of self-determination and avoiding non-maleficence in prognosis disclosure contains a dilemma. First, if patients desire prognosis disclosure that would likely cause psychological pain, this is problematic; a typical example of this is when the physician’s prognosis is less optimistic than the patient’s expectations. Second, when patients refuse prognosis disclosure based on their right not to know, this can be problematic as well, as prognosis disclosure is often required for good outcomes in clinical practice. This is true for cases in which disclosing the prognosis leads to the provision of appropriate palliative care. Unfortunately, prognosis disclosure strategies based solely on self-decision making can neither address nor resolve these two challenges.

## 4. Kantian Autonomy and the Patient’s Best Interest

Stahl and Tomlinson (2017) characterize respect for autonomy as “the capacity of people to make considered choices in light of the facts in order to achieve their values and goals, not merely their expression of a preference [5].” Patient autonomy as presented here does not mean preference-based self-decision making, but rather deliberative choice based on knowledge. We will call this position a prognosis disclosure policy based on Kantian autonomy [16]. This position avoids the dilemma of a prognosis disclosure policy based on self-decision making: Kantian autonomy requires subjects to act on the basis of the practical reason. 

The Kantian approach to autonomy resolves the perceived conflict between respect for the right of self-determination (the right to refuse information) and the principle of non-maleficence (the obligation to avoid harm in the disclosure). Stahl and Tomlinson (2017) further argue that the Kantian approach to autonomy resolves the conflict between the right of self-determination (the right to refuse information) and the principle of beneficence (promotion of patient well-being) [5]. The right not to know one’s prognosis is unacceptable because it distorts the rational choices of patients, ultimately harming their interests.

According to this theory, prognosis disclosure is desirable because it helps patients make deliberative choices about their own treatment, which promotes the right of self-determination. This type of rational choice also seems to accommodate the best interest of patients. In fact, healthcare professionals failing to share prognoses with their patients can result in continuation of futile treatment and delays in receiving palliative care [17], and end-of-life care that patients may otherwise desire may not be provided. Rational choices based on prognosis disclosure allow patients to avoid such situations. The prognosis disclosure policy based on Kantian autonomy aims to achieve a more desirable well-being beyond the harm.

## 5. Remaining Questions

Realizing the value of the Stahl and Tomlinson’s (2017) ideological and intellectualistic analysis of the prognostic disclosure policy based on Kantian autonomy requires a consideration of temporality and the patient’s relationships with others. Even if the prognostic disclosure policy based on Kantian autonomy could theoretically avoid the two dilemmas, it creates another dilemma, i.e., that few people can actually implement it. This new dilemma is revealed in prognosis disclosure just before death.

The Kantian autonomy-based prognosis disclosure policy places a great deal of value on loyalty to truth. Accordingly, even if a patient likely has only 24 h left to live, this policy would have physicians communicating this to their patients once they recognize it. Admittedly, many patients would have already lost consciousness 24 h from death, but this is not the case for all, and some patients remain conscious until the moment of death. Vince and Petros (2006) use the example of an end-stage pediatric patient whose death prognosis is given after the patient required ventilation and sedation due to severe lung disease [18]. Some of the medical team argued that, prior to sedation, the child was competent and, therefore, sedation should be lifted and the patient told of the parents’ decision to withdraw life-sustaining treatment, whereas family members thought that it was too cruel to tell the child. A key feature of this case was the fact that, while the patient acquiesced to sedation, the family and medical team did not discuss the possibility of death with this patient prior to this ventilation and sedation [19]. When death is imminent, is it morally right to remove the child from sedation in order to inform them that their treatment will be discontinued? Without a prior conversation about the potentiality of death and the wishes of the patient in the event of that outcome, it seems absurd to awaken anyone in order to inform them of a decision on death when there are no viable treatment options. Vince and Petros consider whether the desire to provide the patient an opportunity to “put his affairs in order” by making what decisions he could, e.g., whether, and if so how, to say goodbye to his parents, justified lessening the sedation to engage directly with the patient. The prognosis disclosure policy based on Kantian autonomy cannot capture the cruelty that family members inevitably feel about truth telling under these circumstances.

## 6. Clinical Turning Points and Personality: On the Cruelty of the 24 h Death Prognosis Disclosure

As conceptualized, the prognosis disclosure policy based on Kantian autonomy lacks consideration of both temporality and relationships. With regard to prognosis disclosure, consideration of human temporality according to disease progression is essential. For example, for a patient with recurrent cancer, the timing of prognosis disclosure can be divided into three stages depending on the clinical turning point. The first stage is when cancer recurrence is diagnosed and treatment is initiated (prognosis in years), the second is when active treatment methods have been exhausted (prognosis in months), and the third is when death is imminent (prognosis in days). We suggest the prognosis disclosure policy based on Kantian autonomy is effective only up to the second stage; by the third stage, the dilemma of prognosis disclosure becomes apparent. 

One could argue the Kantian justification for prognosis disclosure is no longer useful when there are no viable treatment options and death is imminent. A reason is needed in order for the prognosis to be disclosed in the third stage (i.e., just before death). One reason for prognosis disclosure just before death is that the patient wants to know. Other justification may be possible, e.g., if disclosure contributes to the achievement of expressed goals of the patient or is in concordance with the patient’s values. Regardless of whether a patient has a few days left in their life, or even 24 h left, they may be able to accomplish matters of importance in the time they have remaining. For example, patients may be able to convey important information to family members, as a will of sorts. They may also be able to express appreciation to those who have taken care of them in their lives.

The third stage (i.e., just before death) is more complicated. Family members and healthcare professionals must interpret patient statements in the context of the patient’s whole life. Although it conflicts with the prognosis disclosure policy based on Kantian autonomy, it may be acceptable to withhold prognosis disclosure during this stage, as was decided in the pediatric case presented by Vince and Petros (2006) [18]. We extend this argument to apply to adult patients as well. We argue that principles-based, stereotyped judgment regarding prognosis disclosure may be insufficient especially just prior to the patients’ death. Rather, we find that prognosis disclosure just before death often requires a situational judgment, while taking into account the contextuality of the case and the uniqueness of it. What is important is consideration of the patient’s temporality and relationships with loved one and the medical professionals, as detailed in the next section. Based on this consideration, prognosis disclosure may or may not be performed immediately before death. Whether the decision is made to disclose the prognosis or not, the primary focus should be on maintaining an appropriate care team relationship between the family and health care professionals surrounding the patient. In doing so, the patient’s interests must be maximized in the relationship between the patient, family, and healthcare professionals. This good care relationship should lead to good decisions regarding prognostic disclosure.

## 7. Moralistic Prognosis Disclosure

To complement the prognosis disclosure policy based on Kantian autonomy, we present the moralistic policy for prognosis disclosure. We suggest it as fundamental principles of prognosis disclosure. The moralistic policy for prognosis disclosure is compatible with prognosis disclosure based on Kantian autonomy up through the second stage. However, in the third stage, we would argue that a moralistic policy for prognosis disclosure replaces Kantian autonomy-based prognosis disclosure. To add to the plurality of fundamental principles, we maintain that there is no sole fundamental principle in prognosis disclosure, but rather a combination of competing principles, which may or may not coincide at times along the clinical timescales. The situation changes every moment, depending on the timescale. Depending on the combination of psychosocial and contextual factors in a real-world ethical situation, multiple principles may exert different moral weights. 

Prognosis disclosure based on Kantian autonomy is a theory that is too universal and intellectualistic to be of practical use, especially in the third stage. Some humans are ambivalent about knowing when they will die. Some believe a person can live a more meaningful life by adjusting the purpose of their life and the means of achieving it if they know the approximate time of their inevitable death. Some fear facing death and superimpose the possibility of achieving one’s life purposes on the uncertainty of the future. The ambivalence and variety of perspective are crucial details about human nature that must be acknowledged in order to have a meaningful discussion about prognosis disclosure in the medical setting. Prognosis disclosure based on Kantian autonomy misunderstands or overlooks this aspect of human nature.

In contrast, moralistic policy for prognosis disclosure is situation-dependent and individualistic. François de La Rochefoucauld, a French moralist, is famous for his maxim, “neither the sun nor death can be looked at steadily” [20]. The moralistic viewpoint requires recognition of human weakness and fragility and considers humanity in terms of concreteness and individuality. We argue that the Kantian autonomy-based prognosis disclosure policy is a prima facie duty and must be complemented by the moralistic perspective to which we refer. What is important here is that the human temporality and our relationships with others are secured in prognosis disclosure.

For the community of patients, family members, and physicians affected by this, it may be cruel to inform patients explicitly that they will die in the next 24 h. It has traditionally been explained that prognosis disclosure just before death is unlikely to increase significantly the patient’s remaining quality of life; it also seems cruel because it may cause pain to patients with insufficient time to mitigate that pain. The prognosis disclosure policy based on Kantian autonomy values the truth that lies beyond that pain. However, the moralistic point of view that we present in this paper is different from both the Kantian account and the traditional account; in the latter, voluntary self-decision making potentially conflicts with non-maleficence toward patients. Truth is certainly important; however, in prognosis disclosure during the third stage (just before death), the value of truth telling must be congruent with a firm consideration of the patient’s values and humanity. It does not mean only considering the psychological pain (of the patients themselves). Rather, the root of the cruelty in prognosis disclosure lies in the fact that patients themselves may be alienated from the network of relationships, such as those with family members and healthcare professionals. Relationships and historicity are essential to the person, and prognosis disclosure must accommodate the individual patient; decisions must be informed by a patient-specific narrative. The 24 h prognosis disclosure may disrupt these relationship and historicity, yielding a feeling of cruelty. If the historicity of this network of relationships is not taken into account and prognosis disclosure is based on mere (Kantianistic) ideology, it would mean the collapse of this network of relationships. Historicity in this context is the accumulation of time shared by the patient, the family, and the health care professionals who have provided care, and the narratives developed over that time. Historicity shapes the patient’s personality in the network of relationships. Without respect for this historicity, any decision about the patient is meaningless. To disrupt this network of relationships and send the patient off into another world alone would cause the patient emotional distress, but more than that, it would be cruel in the sense that the patient’s historic personality would be disregarded. In order to avoid this cruelty, it would be necessary to adopt a flexible approach that emphasizes the values of patients at the forefront.

## 8. Conclusions

The ethical discourse on prognosis disclosure is not yet well established. Although the West and East show cultural differences in prognosis disclosure, particularly with regard to patient awareness, they share in common the acknowledgment that prognoses are not actually disclosed enough in advance to satisfy patient wishes. The prognosis disclosure policy based on Kantian autonomy demands truth telling under all circumstances. However, the application of the Kantian point of view must take into account the clinical timescale specific to the patient. Certainly, the introduction of the Kantian point of view is advantageous when the prognosis is expected to remain somewhat predictable (first and second stage). However, we must hesitate to introduce the Kantian point of view in the just before death (third stage). That is because the intellectualistic view of humans that underlies this policy impairs the social relationships patients have with those around them. All human beings inherently possess temporality and relationships with others. Relationships between patients, family members, and healthcare professionals, which have been formed uniquely and concretely until the patient approaches their end of life, share this temporality, and the patient’s values are formed by such relationships. We call this the moralistic perspective. Prognosis disclosure must always be unique in order for the patients living in the final stages of their life to be authentically autonomous. In other words, the decision to disclose a prognosis or not can only be determined by the relationships fostered over time with the patients. 

One significance of the present study is its demonstration of how to balance a theory and a practice pertaining to the ethical discourse on prognosis disclosure. The policy based on Kantian autonomy presented by Stahl and Tomlinson (2017) represents groundbreaking ethical discourse on prognosis disclosure. This paper supplements their theory by adding a moralistic perspective that accommodates the clinical stage of the patient prior to death, allowing the ethical discourse on prognosis disclosure to be taken a step further.

## Data Availability

Not applicable.
